# Postoperative analgesia in total knee arthroplasty for posttraumatic arthritis: A randomised controlled trial comparing patient‐controlled intravenous analgesia, continuous femoral/sciatic nerve block and continuous local infiltration analgesia

**DOI:** 10.1002/jeo2.70599

**Published:** 2025-12-28

**Authors:** Bastian Mester, Tobias Ohmann, Christine Seelmann, Anne Terschluse, Claas Güthoff, Christian Gerach, Nikolaus Brinkmann, Marcel Dudda

**Affiliations:** ^1^ Department of Trauma, Hand and Reconstructive Surgery University Hospital Essen Essen Germany; ^2^ Research Department BG‐Klinikum Duisburg Duisburg Germany; ^3^ University of Duisburg‐Essen Universitätsstraße 2 Essen Germany; ^4^ Centre for Clinical Research BG‐Klinikum Unfallkrankenhaus Berlin Berlin Germany; ^5^ Department of Anesthesiology and Intensive Care Medicine BG‐Klinikum Duisburg Duisburg Germany; ^6^ Department of Orthopaedics and Trauma Surgery, BG‐Klinikum Duisburg University of Duisburg‐Essen Duisburg Germany

**Keywords:** local infiltration analgesia, nerve block, pain management, posttraumatic arthritis, total knee arthroplasty

## Abstract

**Purpose:**

Postoperative pain remains a major obstacle to early mobilisation following total knee arthroplasty (TKA) and is particularly challenging in patients with posttraumatic arthritis due to prior injury, scarring, and complex surgeries. While various analgesic strategies have been evaluated in degenerative TKA, evidence in posttraumatic cases is lacking. This study is the first to compare patient‐controlled intravenous analgesia (PCIA), continuous femoral/sciatic nerve block (cFNB/cSNB), and continuous local infiltration analgesia (cLIA) in this specific population.

**Methods:**

In this prospective, monocentric, randomised controlled trial, 92 patients undergoing TKA for posttraumatic arthritis were allocated to PCIA (*n* = 31), cFNB/cSNB (*n* = 30) or cLIA (*n* = 31). Postoperative pain was assessed using the numeric rating scale (NRS) from day 1 (t1) to discharge (t6). Secondary outcomes included range of motion (ROM), rescue analgesic use, Knee Society Score (KSS) and patient satisfaction. Statistical comparisons between groups were performed using adjusted pairwise tests.

**Results:**

At t1, patients in cFNB/cSNB reported significantly lower pain scores compared to cLIA (*p* = 0.039). cLIA required significantly more rescue analgesics at t1/t2 (both *p* < 0.05). Active/passive knee flexion was consistently higher in cFNB/cSNB from t1 to t6 (all *p* < 0.05). No differences were observed in postoperative mobility, KSS or satisfaction. All groups showed significant improvement in pain and ROM over time.

**Conclusions:**

In patients undergoing TKA for posttraumatic arthritis, cFNB/cSNB provided superior early postoperative pain control and functional recovery compared to PCIA and cLIA. The findings suggest that standard fast‐track protocols may not be fully applicable in this complex patient population, highlighting the need for individualised analgesic strategies.

**Level of Evidence:**

Level I.

**Abbreviations**
CC: condylar‐constraint
cLIA: continuous local infiltration analgesia
cNFB/cSNB: continuous femoral and sciatic nerve blockade
CONSORT: consolidated standards of reporting trials
CPM: continuous passive motion
CR: cruciate‐retaining
EPZ: endoprothesenzentrum
ICU: intensive care unit
KSS: Knee Society Score
LIA: local infiltration analgesia
NRS: numeric rating scale
PCIA: patient‐controlled intravenous analgesia
PS: posterior‐stabilised
ROM: range of motion
SD: standard deviation
TKA: total knee arthroplasty
WHO: World Health Organization

## PURPOSE

Major orthopaedic surgery in general and Total Knee Arthroplasty (TKA) in special are known to cause relevant postsurgical pain levels, especially within the first days after surgery [16, 33]. The maximum pain level is reached within the first three to six hours after the operation [4, 16]. At the same time, up to 20% of the patients are reported to be unsatisfied with the postoperative result [17, 34, 41]. Patient's expectations before surgery, the degree of improvement in knee function and pain relief following surgery were identified as the key factors for dissatisfaction after TKA [14, 17].

Postsurgical pain can delay mobilisation and impair functional outcome after TKA [51]. Therefore, pain relief, especially in the early postoperative phase after TKA is essential to achieve satisfying functional results.

In the past, the management of postsurgical pain after TKA has been discussed extensively. Different analgetic tools and their combination in terms of multimodal concepts have been described to be effective [26, 48]. However, evidence regarding the efficacy and optimal combinations of interventions varies widely across available studies [45]. Peripheral nerve blockades are widely accepted as the standard for the treatment of postoperative pain after TKA [48]. Due to the neural supply of the knee joint, continuous femoral and sciatic nerve blockade (cNFB/cSNB) is commonly used. However, quadriceps muscle strength can be affected, which limits postoperative mobilisation and increases the risk of falls [9]. Patient‐controlled analgesia administering intravenous opioids (PCIA) is also a safe and effective tool in the management of moderate to severe pain after TKA [13, 50]. As the handing over of responsibility into the patient's hands seems favourable, it is associated with adverse events including nausea and vomiting, respiratory depression and urinary retention [26, 44]. Obviously, incidence of these events may influence patient's satisfaction and postoperative functional results [48]. Perioperative local infiltration analgesia (LIA) has gained increasing importance in the setting of postoperative pain management after TKA, especially in the context of fast‐track concepts [25, 26, 49]. According to literature, LIA after TKA provides satisfying pain reduction and can reduce additional opioid consumption in the very early postoperative phase [15, 52, 54]. On the other hand, rebound pain after 48 to 72 hours due to the short duration of action may impede mobilisation [53].

In the last years, application systems for continuous application of slow‐acting local anaesthetics (continuous local infiltration analgesia, cLIA) over a reservoir pump have been developed and clinically implemented [8], aiming to take all the advantages of LIA and solve the problem of rebound pain. In the setting of postoperative pain management after TKA, different authors could demonstrate good to excellent results regarding pain reduction, opioid consumption, quadriceps motor function and falls, at least equivalent to the effect of cNFB/cSCB [23, 25, 46, 55].

TKA for posttraumatic arthritis must be considered a different entity and represents a challenging task in arthroplasty. Joint instabilities, malalignment, bone defects, non‐union and scarring due to multiple earlier operations make soft tissue preparation and endoprosthesis implantation technically more demanding [6, 12, 18, 30]. Regularly, the choice of higher levels of constraint and stems is unavoidable. At the same time, patients are younger on average with a higher risk for complications and revision surgery in the future [7, 10, 28, 31, 36, 37, 41, 43, 47].

The functional results of TKA for posttraumatic arthritis do not compare with primary TKA for degenerative arthritis [2, 19, 20, 28, 29, 38, 39, 47]. It can be deducted from the abovementioned data that peri‐ and postoperative pain management itself is more challenging in TKA for posttraumatic arthritis, as patient‐related outcome measures, including pain scores, are well‐known to be worse than in TKA for primary osteoarthritis [28]. Subsequently, postoperative pain management is expected to be more difficult.

The efficacy of cLIA as pain management modality after TKA for primary osteoarthritis has been proven in the past [23, 25, 45], and other modalities like PCIA and cFNB/cSNB have been elaborated extensively [15, 22, 45, 49]. However, the significance of cLIA in comparison to established analgetic means like PCIA and cFNB/cSNB in the context of TKA for posttraumatic arthritis remains unclear. On the other hand, TKA due to posttraumatic conditions is becoming increasingly common, especially in patients before the age of 50 years [36].

To the best of our knowledge, this is the first study to compare pain reduction after TKA for posttraumatic arthritis by PCIA versus cFNB/cSNB versus cLIA and its influence on knee function and patient's satisfaction in the early postoperative phase.

## METHODS

### Trial design and participants

This prospective, monocentric, randomised clinical trial included patients with posttraumatic knee arthritis undergoing primary TKA. After applying exclusion criteria, 92 patients were randomised to receive PCIA (n = 31), cFNB/cSNB (*n* = 30), or cLIA (*n* = 31). Pain was assessed on a numeric rating scale (NRS) from postoperative day 1 (t1) to discharge (t6). Active/passive ROM, leg lift, rescue analgesic use, mobility, satisfaction, and KSS were recorded.

Patients diagnosed with posttraumatic arthritis of the knee who subsequently underwent primary TKA in a local centre for endoprosthesis (‘Endoprothesenzentrum’, EPZ) as well as a level‐one trauma centre, were assessed for eligibility. Posttraumatic arthritis was defined as clinical symptoms and radiological degenerative alterations following an osseous and/or ligamentous injury of the knee joint in the past, including fractures of the distal femur and proximal tibia with involvement of the joint surfaces, cruciate and collateral ligament injuries, traumatic chondral damage or meniscal tears.

Partial knee arthroplasty due to unicompartimental arthritis, isolated femoropatellar joint replacements and patient‐individualised partial arthroplasty were excluded, as well as revision TKA, prosthesis component changes and isolated soft tissue revisions. Further exclusion criteria were general contraindications to the application of the analgetic treatment regimens, patient's rejection to participate, a planned postoperative stay on the intensive care unit (ICU) due to patient's pre‐existing conditions, advanced dementia and mental inability to comply. Postoperative changes of the given analgetic regimen, an unplanned ICU stay and relevant complications within the observation period requiring early revision surgery (infection, component changes) led to exclusion during the study (dropout).

**Figure 1:**
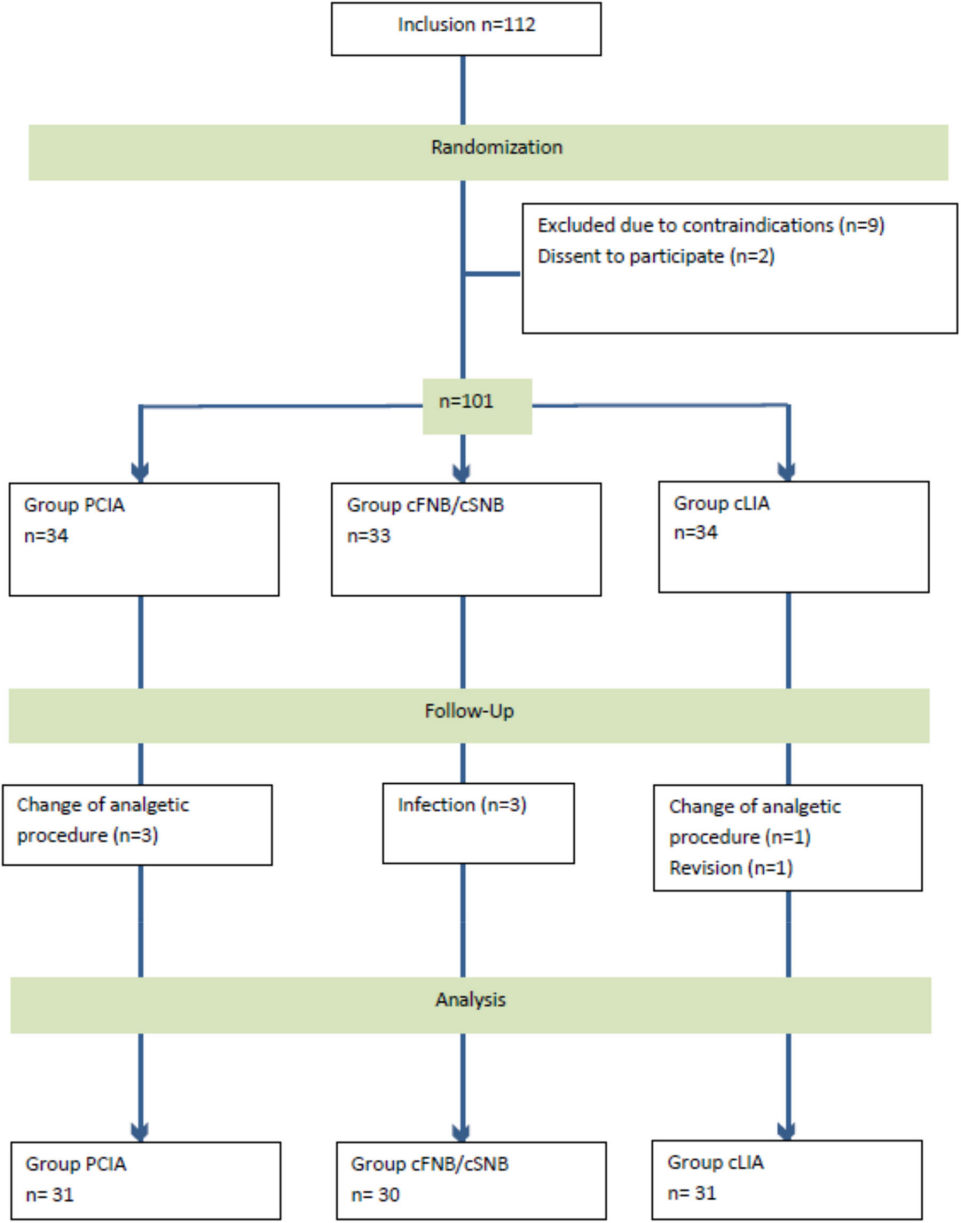
Flow of patients through the study. CONSORT diagram presenting the enrolment of the final study population according to [40].

The patient's enrolment process and application of inclusion and exclusion criteria are shown in Figure 1.

This investigation was conducted in accordance with the consolidated standards of reporting trials (CONSORT) guidelines [40] and the principles of Good Clinical Practice, as well as with the study protocol and the statistical analysis plan. The protocol was reviewed and approved by the ethics committee of the University of Witten/Herdecke (No. 19/2016) and did not change during the study. The trial was registered at the German Clinical Trials Register (No. DRKS00012500).

### Intervention

Anaesthesia protocol was similar for all patients of the study population. Postoperatively, the patients received one of three analgesic treatment regimens according to the randomisation protocol.

In group PCIA, a peripheral venous catheter was placed and a standard piritramide solution (Dipidolor® 7.5 mg/ml; Janssen‐Cilag GmbH, Neuss, Germany) was administered over an infusion pump. An initial bolus of 3 mg was applied. The lockout interval was set to 12 minutes, with a maximum dosage of 12 mg/hour. The PCIA was discontinued on day 3 after the operation.

For patients of group cFNB/cSNB, continuous peripheral nerve catheters were brought in by the anaesthetist adjacent to both the femoral and sciatic nerve (PlexoLong, Pajunk®, Geisingen, Germany). Positioning was controlled by sonography. A solution of ropivacaine (20 ml ropivacaine 0,75% (Ropivacain Fresenius® 2 mg/ml; Fresenius Kabi, Kriens, Switzerland) for cFNB, 15 ml ropivacaine 0,75% cSNB) was applied over an infusion pump intraoperatively, offering a continuous flow of 8 ml/h (ropivacaine 0.2%). Both cFNB and cSNB were removed three days after the operation.

In group cLIA, an intraarticular catheter (InfiltraLong 19 G x 500 mm, 75 mm; Pajunk®, Geisingen, Germany) was placed by the surgeon intraoperatively before wound closure via a split cannula adherent to the joint capsule. An affiliated elastomeric infusion pump (InfiltraLong Fuser Pump 500; Pajunk®, Geisingen, Germany) was connected to the catheter, filled with 350 ml ropivacaine solution (Ropivacain Fresenius® 2 mg/ml; Fresenius Kabi, Kriens, Switzerland). Another 50 ml of ropivacaine was injected intraoperatively into the joint capsule and subcutaneous tissue in a circular manner. The elastomeric pump provided a continuous flow of 8 ml/hour over approximately 44 h. The catheter was consecutively removed.

Irrespective of the group‐specific analgetic treatment, all patients received a peroral baseline analgesia corresponding to a standard postoperative application protocol. It contained ibuprofene (Ibu‐ratiopharm® 3 × 600 mg; Ratiopharm, Ulm, Germany), etoricoxibe (Arcoxia® 1 × 90 mg; Grünenthal, Aachen, Germany) or novaminsulfone (Novaminsulfon‐ratiopharm® 4 × 500 mg; Ratiopharm, Ulm, Germany), depending on allergies or gastrointestinal, cardiocirculatory and other individual contraindications. In case of insufficient analgesic efficacy (rest pain NRS > 3, motion pain NRS > 5), rescue analgesics of higher potency were available, and application was documented: buprenorphine (Temgesic®0.2 mg sublingual; Indivior, Dublin, Ireland), piritramide (Dipidolor® 7,5 mg rapid infusion intravenous; Janssen‐Cilag GmbH, Neuss, Germany), oxycodone (Oxygesic akut® 10 mg peroral; Mundipharma, Frankfurt, Germany and oxycodone/naloxone (Targin® 10/5 mg peroral; Kohlpharma, Merzig, Germany).

### Surgery

The TKA surgery was performed by two experienced lead surgeons of the local centre for endoprosthesis (Endoprothesenzentrum, EPZ) in accordance with the Endocert® standards, Berlin, Germany. All patients underwent general anaesthesia and received perioperative intravenous antibiotic prophylaxis. Patients were positioned supine, and a tourniquet was applied. Access to the knee joint was achieved via a standard anteromedial approach with parapatellar arthrotomy; skin incisions were adapted if previous surgical scars were present. Dependent on ligamentous instability, malalignment, bony defects and bone quality, the prosthesis type, level of constraint, and the use of femoral and/or tibial stems were selected based on preoperative digital planning and intraoperative assessment. The choice of constraint included cruciate‐retaining (CR), posterior‐stabilised (PS), condylar‐constraint (CC) and hinged designs, in terms of an individualised approach to the preoperative functional conditions and surgical requirements of each patient. In this study, endoprostheses from different manufacturers were used: Triathlon® Knee System (Stryker, Kalamazoo, USA), Legion® Total Knee System (Smith&Nephew, Watford, United Kingdom), ATTUNE® Knee System (DePuy Synthes, Warsaw, USA) and MUTARS GenuX® (Implantcast, Buxtehude, Germany).

The first dressing change and drain removal were performed on the first postoperative day. All patients received antithrombotic prophylaxis in the course of the postoperative treatment. Postoperative X‐rays of the knee in two planes, routine blood sampling, and regular clinical follow‐ups were conducted. Physiotherapy was initiated once daily, with full weight‐bearing and an allowed range of motion of extension/flexion 0/0/90°, supported by a standard continuous passive motion (CPM) device.

### Outcome – primary endpoint

The primary endpoint for the three groups was the postoperative pain reduction according to a semiquantitative numeric rating scale (NRS) on the first day after surgery (t1). The scale ranged from ‘0’ ( = no pain) to ‘10’ ( = maximum pain level), and the pain level was assessed once a day under physical strain/physiotherapy.

### Outcome – secondary endpoints

Secondary endpoints were the postoperative pain reduction according to the NRS on t2 to t6. Additionally, the demand for Rescue Analgesics was documented and every single dosage was counted. Further secondary endpoints included range of motion (ROM), postoperative mobility, the Knee Society Score (KSS) and patient's overall satisfaction.

Passive ROM in terms of knee flexion (degrees) was measured according to the neutral‐zero method by medical goniometry. Furthermore, the ability to lift the extended leg (yes/no) against gravity was queried.

Patient's postoperative mobility was assessed once a day and categorised in ‘none’, ‘standing in front of the bed’, ‘walking in the room’, ‘walking on the floor’ and ‘going stairs’.

The KSS is a simple scoring system to rate the patient's functional abilities before and after total knee arthroplasty [21]. The KSS is a two‐part score that consists of a ‘Knee Score’ section (seven items) and a ‘Functional Score’ section (three items). Both sections are scored from ‘0’ to ‘100’ with lower scores being indicative of worse knee conditions and higher scores being indicative of better knee conditions.

Finally, the patient's satisfaction with the operative treatment was evaluated before discharge from hospital by means of an NRS. The scale ranged from ‘0’ (= no satisfaction) to ‘10’ (= maximum satisfaction).

**Figure 2:**
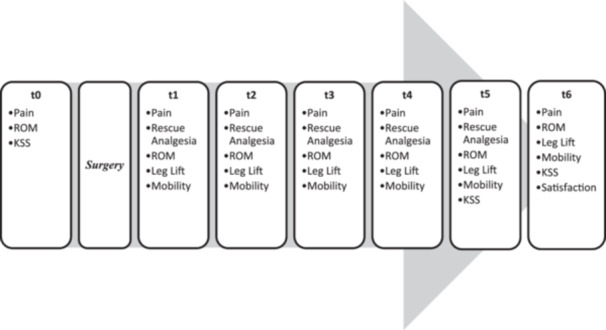
Investigation time points t0–t6. Time points before and after the operation (OP) in the course of the study, with the respective outcome parameters collected. KSS, Knee Society Score; ROM, range of motion.

Figure 2 shows the outcome measures at the examination time points t0 to t6.

### Randomisation

The computerised randomisation sequence was created by an independent person. Recruiting, baseline examination (t0) and randomisation of the participants were done one day before surgery (t0) by study staff. Randomisation was conducted by means of the closed envelope method.

Blinding was suspended immediately after randomisation and after obtaining patients' informed consent to participate in this investigation. Immediate suspension of blinding was carried out to communicate instructions for handling to the application of the fundamentally different analgetic regimens in the initial postoperative phase.

The group membership was documented in the patient's records.

### Statistical analysis

Absolute and relative frequencies and means with standard deviations were used for the sample description of outcome parameters. Hypotheses were tested with Kruskal–Wallis H Tests with subsequent Dunn's Tests for Bonferroni‐adjusted pairwise comparisons in case of significance or with Fisher's Exact Tests. *p* Values > 0.05 were considered significant.

All analyses were conducted with SPSS 27.0 (IBM SPSS Statistics for Windows, Version 27.0. Armonk, NY; IBM Corp).

### Patient and public involvement

Patients were not involved in designing the research question, outcome measures, or interpretation or writing up of the results of this study. Patient representatives in our ethics committee were asked for comments on general comprehensibility.

The patient representatives of the hospital were informed about the study and its start. Results will be presented to patients and the public as part of regular information events.

## RESULTS

### Demographic data

Due to contraindications against one of the analgetic treatment options or patient's dissent to participate, *n* = 11 patients were initially excluded. Postoperatively, in the cNFB/cSNB group, there was a dropout of *n* = 3 patients because of deep wound infections, of *n* = 1 patient in the cLIA group because of revision surgery (inlay revision) and of *n* = 3 patients in the PCIA and *n* = 1 in the cLIA group because of unplanned change of the postoperative analgetic regimen, respectively.

After application of exclusion criteria, *n* = 92 patients (female=25, male=67; mean age 58.08 ± 9.46 years, min 29, max 80) were included in this investigation for final analysis.

Participants' baseline demographic data and information regarding the initial trauma as well as the surgery (TKA) are summarised in Table 1.

**Table 1:** Demographic patient data.

	Total (92)	PCIA (*n* = 31)	cFNB/cSNB (*n* = 30)	cLIA (*n* = 31)
Sex, *n* (%)				
Male	67 (72.8%)	20 (64.5%)	24 (80%)	23 (74.2%)
Female	25 (27.2%)	11 (35.5%)	6 (20%)	8 (25.8%)
Age, mean ± SD	58.1 ± 9.5 (29–80)	60 ± 7 (48–71)	58 ± 11 (29–79)	56 ± 10 (32–80)
BMI (kg/m^2^), mean ± SD (range)	30.54 ± 6.2 (19.1–53.4)	29.5 ± 5.8 (19.1–44.1)	30.7 ± 6.5 (22–53.4)	31.5 ± 6.9 (19.7–43.8)
Smoking (yes), *n* (%)	29 (31.5%)	13 (41.9%)	9 (30%)	7 (22.6%)
Type of injury, *n* (%)				
Fracture	37 (40.2%)	15 (48.4%)	12 (40%)	10 (32.3%)
Menisco‐Ligamentous	36 (39.1%)	8 (25.8%)	15 (50%)	13 (41.9%)
Other	19 (20.7%)	8 (25.8%)	3 (10%)	8 (25.8%)
Number of previous surgeries, mean ± SD (range)	3.6 ± 3.3 (0–16)	4 ± 3.5 (0–16)	3.1 ± 2.7 (0–13)	3.4 ± 2.7 (0–15)
Interval trauma – TKA (months) mean ± SD (range)	211.2 ± 183.6 (2–721)	218.7 ± 193.9 (3–585)	203.5 ± 206.8 (2–721)	210.5 ± 152.1 (9–441)
Inpatient treatment period (days), mean ± SD (range)	13.2 ± 5.3 (8‐42)	13 ± 6.4 (9‐42)	13.2 ± 4 (8‐26)	13.5 ± 5.4 (9‐28)
Operation duration (minutes), mean ± SD (range)	143.7 ± 46.5 (70‐286)	143 ± 49.1 (70‐286)	145.5 ± 50 (72‐265)	142.4 ± 41 (85‐250)
Choice of constraint, *n* (%)				
CR	36 (39.1%)	12 (38.7%)	9 (30%)	15 (48.4%)
PS	20 (21.7%)	9 (29%)	9 (30%)	2 (6.5%)
CC	32 (34.8%)	8 (25.8%)	11 (36.7%)	13 (41.9%)
Hinged	4 (4.3%)	2 (6.5%)	1 (3.3%)	1 (3.2%)

*Note*: Additional information regarding the initial trauma, intervals and data of the index operation.

Abbreviations: CC, condylar‐constraint; CR, cruciate‐retaining; PS, posterior‐stabilised; SD, standard deviation; TKA, total knee arthroplasty.

The overall mean time interval between trauma and operation was 211.18 ± 183.64 days (min 2, max 721). 44.6% (*n* = 41) had a fracture of the lower extremity as a result of the initial trauma; 80.5% of these cases were tibial head fractures. Injuries of the meniscus and ligament tears without bony lesions were reported in *n* = 39 cases (42.4%). On average, study patients had 3.54 ± 3.04 (min 0, max 16) operations on the affected limb following the initial trauma before TKA.

Regarding the index operation (TKA), in *n* = 36 the level of constraint was CR, in *n* = 20 PS and *n* = 32 CC. Four patients received a hinged knee prosthesis.

Presenting with a mean BMI of 30.55 ± 6.39 (min 19.05, max 53.42), the study collective suffered from obesity according to the World Health Organization (WHO) definition [11]. 28.3% (*n* = 26) reported pre‐existing chronic pain before TKA with regular pain medication for different indications than pain of the affected knee.

### Primary endpoint

**Figure 3:**
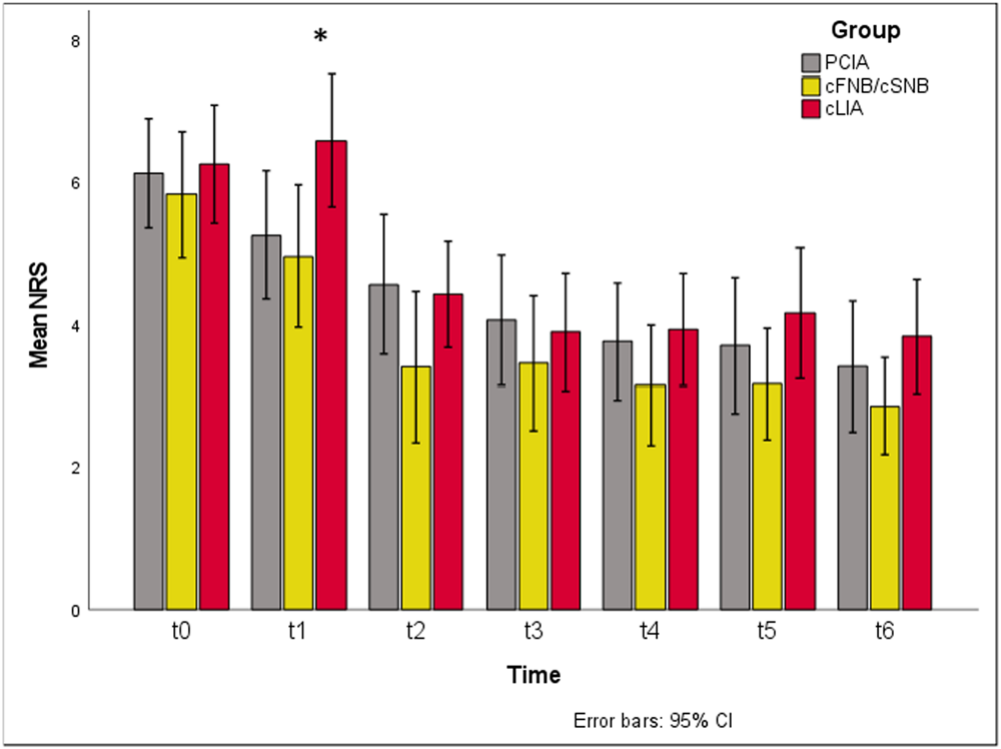
Pain reduction over time and comparison between study groups. Significant improvement over time for all three groups from t1 to t6; significantly less pain for cFNB/cSNB compared to cLIA at t1 (*p* = 0.039 overall; cFNB/cSNB versus cLIA *p*_adj_ = 0.049). cFNB/cSNB, continuous femoral and sciatic nerve blockade; cLIA, continuous local infiltration analgesia; cSNB, continuous femoral and sciatic nerve blockade; NRS, numeric rating scale; PCIA, patient‐controlled intravenous analgesia

No significant differences in demographics or surgical parameters were found. At t1, cFNB/cSNB showed significantly lower NRS pain scores vs. cLIA (*p* = 0.039 overall; cFNB/cSNB versus cLIA *p*_adj_ = 0.049; Figure 3).

### Secondary endpoints

Comparing the postoperative pain reduction at t2 to t6 according to the NRS, no statistically significant differences were found between PCIA, cFNB/cSNB and cLIA (n.s.). However, there was a trend towards higher NRS pain scores in CLIA versus cFNB/cSNB with constantly higher absolute NRS values in cLIA than in cFNB/cSNB from t2 to t6, without statistical significance. Results regarding the postoperative pain reduction in the course of the investigation from t0 to t6, including the improvement over time and the significant difference at t1 are shown in Figure 3.

**Figure 4:**
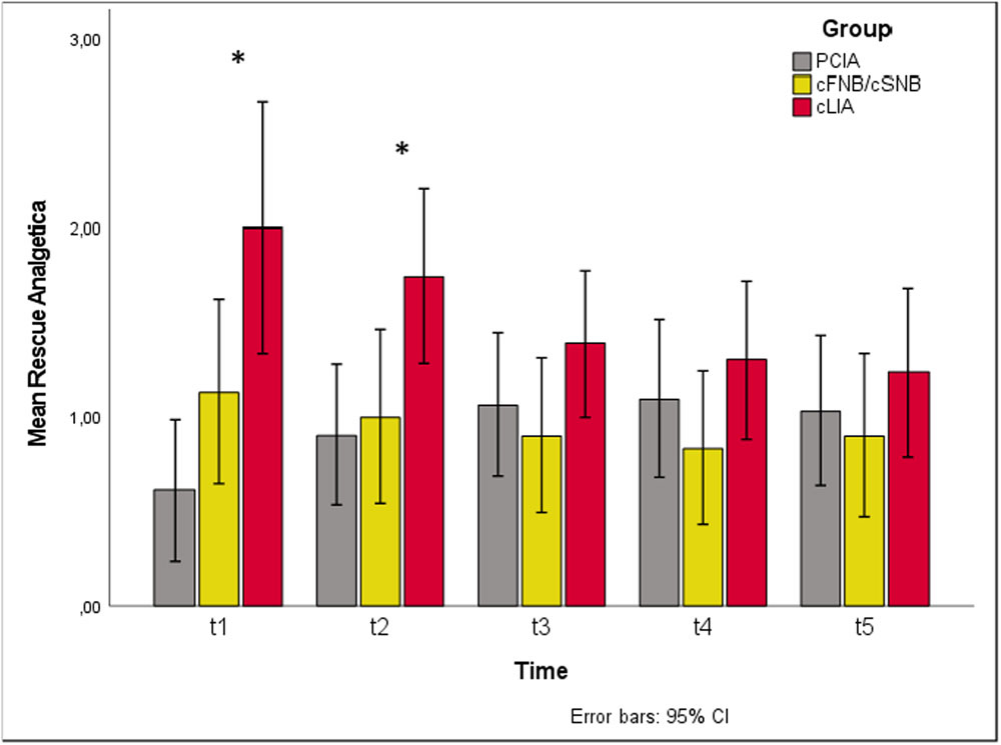
Demand for rescue analgetics over time and comparison between study groups. Significantly higher demand for rescue analgetics at t1 and t2 in the cLIA group compared to the PCIA group (t1: *p* < 0.001, PCIA versus cLIA *p*_adj_ < 0.001; t2: *p* = 0.014, PCIA versus cLIA *p*_adj_ = 0.024). cFNB/cSNB, continuous femoral and sciatic nerve blockade; cLIA, continuous local infiltration analgesia; PCIA, patient‐controlled intravenous analgesia.

At t1 as well as t2, the demand for rescue analgesics was significantly higher in the cLIA group than in the PCIA group (t1: *p* < 0.001, PCIA versus cLIA *p*_adj_ < 0.001; t2: *p* = 0.014, PCIA versus cLIA *p*_adj_ = 0.024; Figure 4).

Comparing the ROM between the three study groups, cFNB/cSNB showed a significantly higher active knee flexion at t1 and t2 compared to other study groups (t1: *p* = 0.011, cFNB/cSNB versus cLIA *p*_adj_ = 0.008; t2: *p* = 0.011, cFNB/cSNB versus PCIA *p*_adj_ = 0.017). Although the differences in active knee flexion remained consistent in terms of higher absolute flexion angles for cFNB/cSNB versus cLIA over the whole investigation period, statistical significance was not reached (n.s.). Passive knee flexion was significantly higher in patients treated with cFNB/cSNB from t2 to t6 (t2: *p* = 0.01, cFNB/cSNB versus PCIA *p*_adj_ = 0.025, cFNB/cSNB versus cLIA *p*_adj_ = 0.028; t4: *p* = 0.043; t5: *p* = 0.044; t6: *p* = 0.023, cFNB/cSNB versus cLIA *p*_adj_ = 0.019).

**Figure 5:**
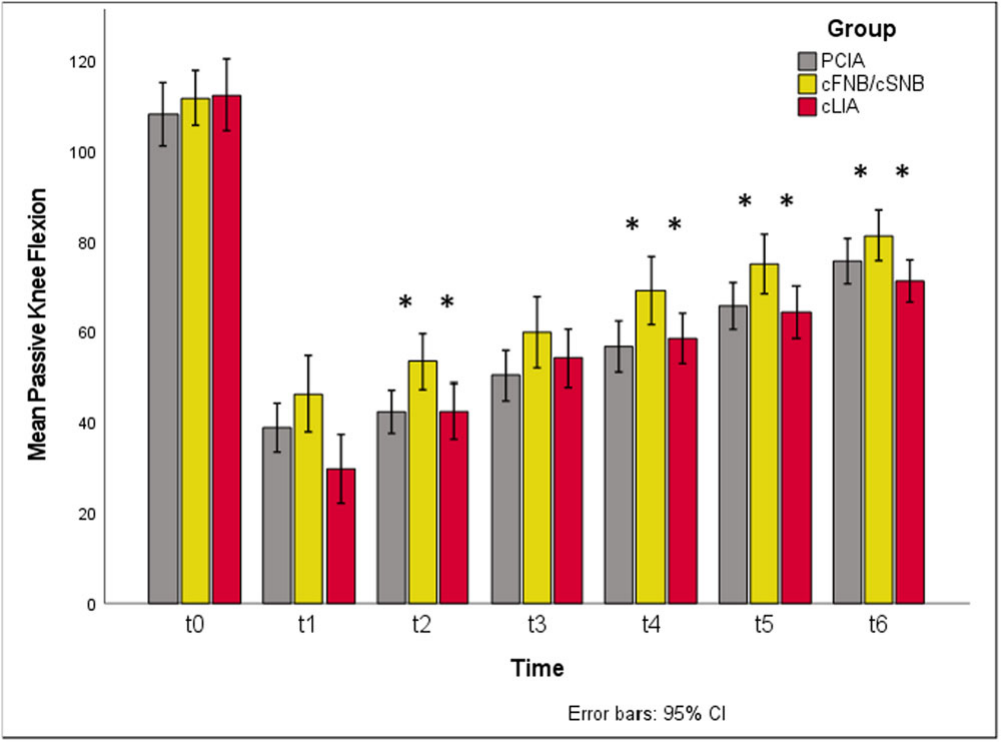
Passive knee flexion over time and comparison between study groups. Significantly higher passive knee flexion for cFNB/cSNB compared to both PCIA and cLIA at t2, t4, t5 and t6 (t2: *p* = 0.01, cFNB/cSNB versus PCIA *p*_adj_ = 0.025, cFNB/cSNB versus cLIA *p*_adj_ = 0.028; t4: *p* = 0.043; t5: *p* = 0.044; t6: *p* = 0.023, cFNB/cSNB versus cLIA p_adj_ = 0.019). cFNB/cSNB, continuous femoral and sciatic nerve blockade; cLIA, continuous local infiltration analgesia; PCIA, patient‐controlled intravenous analgesia.

**Figure 6:**
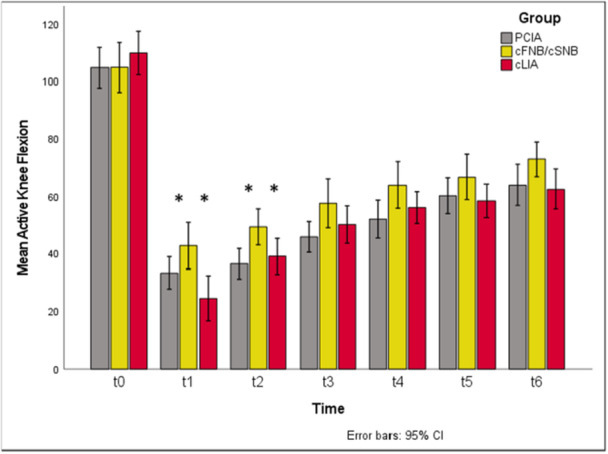
Active knee flexion over time and comparison between study groups. Significantly higher active knee flexion for cFNB/sSNB compared to both PCIA and cLIA at t1 and t2 (t1: *p* = 0.011, cFNB/cSNB versus cLIA *p*_adj_ = 0.008; t2: *p* = 0.011, cFNB/cSNB versus PCIA *p*_adj_ = 0.017). cFNB/cSNB, continuous femoral and sciatic nerve blockade; cLIA, continuous local infiltration analgesia; PCIA, patient‐controlled intravenous analgesia.

Differences between PCIA and cLIA could not be shown (n.s.). Results for ROM, including the group differences of cFNB/cSNB versus PCIA and cLIA are presented in Figures 5 and 6.

The results for PCIA, cFNB/cSNB and cLIA regarding the ability to lift the extended leg against gravity revealed no significant differences, nor regarding postoperative mobility (both n.s.).

On average, it took patients 5.9 ± 1.0 postoperative days to reach mobility on stairs in group PCIA, 4.8 ± 1.2 days in group cFNB/cSNB and 4.9 ± 1.2 days in group cLIA.

Regarding the KSS results, we could not demonstrate a significant difference between the three study groups (n.s.).

The mean final satisfaction with the operative treatment before discharge was 8.62 ± 2.3 for PCIA, 9.0 ± 1.3 for cFNB/cSNB and 8.8 ± 1.4 for cLIA. No significant differences were found for the satisfaction with the operative treatment (n.s.).

## DISCUSSION

This randomised controlled trial is the first to compare three postoperative analgesic strategies ‐ patient‐controlled intravenous analgesia (PCIA), continuous femoral and sciatic nerve block (cFNB/cSNB), and continuous local infiltration analgesia (cLIA) ‐ in patients undergoing TKA for posttraumatic arthritis. Our findings highlight cFNB/cSNB as the most effective approach for early postoperative pain control and functional recovery, showing superior outcomes in pain scores, rescue analgesic demand, and both passive and active ROM.

While all three analgesic modalities improved pain control and knee function over time, only cFNB/cSNB showed significantly lower pain scores on the first postoperative day (t1). A trend towards lower NRS pain scores in cFNB/cSNB versus cLIA, with constantly lower absolute NRS value,s was also seen. Significantly improved passive knee flexion for cFNB/cSNB compared to cLIA could be demonstrated from t1 to discharge (t6) and significantly improved active knee flexion from t1 to t2 with consistently higher active flexion angles for cFNB/cSNB versus cLIA from t3 to t6 (n.s.). These early advantages are particularly relevant, as pain can delay mobilisation and impair rehabilitation. Early postoperative results regarding pain and function may have a certain predictive value for the long‐term outcome of TKA. In a retrospective investigation on n = 196 patients who underwent unilateral TKA for primary osteoarthritis, Oka et al. could demonstrate that early improvement in knee flexion after TKA indicates the likelihood of achievement of satisfying knee flexion angles at 12 months after TKA [35]. Apart from other parameters, early mobilisation, improved flexion mobility and reduced pain scores in the early postoperative phase were identified as ‘game changers’ to achieve consistently optimal long‐term outcomes of TKA, and extend well beyond the expedited hospital discharge [3]. Our results are consistent with previous studies supporting peripheral nerve blocks in TKA [48, 54]. However, most prior evidence focuses on primary osteoarthritis and may not fully apply to posttraumatic cases.

The posttraumatic arthritis cohort in this study represents a particularly challenging population: relatively young, with higher BMI, frequent pre‐existing chronic pain, and a high number of previous surgeries (mean 3.5). These factors are associated with worse outcomes compared to primary osteoarthritis [20, 29, 43]. The average interval between trauma and arthroplasty was over seven months, and intraoperative complexity was high, requiring constrained implants in the majority of patients.

cLIA is commonly promoted in fast‐track protocols as a less invasive alternative [23, 25, 46]. Kutzner et al. conducted a similar randomised trial, evaluating pain intensity, ROM, demand for rescue analgesia and mobility within the first five days after primary TKA in *n* = 120 patients [25]. The comparison of postoperative analgetic regimens of cLIA (group A) and cFNB (group B) showed no differences regarding pain intensity, the demand for rescue analgesia nor ROM. On the other hand, the postoperative mobility was significantly faster reached in the cLIA group. The authors concluded that cLIA was equivalent in analgesic efficacy and potentially superior in early mobilisation.

In contrast, our study found cLIA to be associated with higher pain levels and increased rescue analgesic demand in the early phase. This may relate to insufficient analgesia during the critical first 48 hours postoperatively ‐ especially relevant in posttraumatic knees where tissue scarring and altered anatomy may blunt the effect of intraarticular diffusion. While cLIA has shown equivalence to nerve blocks in primary TKA [25, 52], our results suggest these benefits may not translate to posttraumatic settings.

As depicted above, in the present study both the continuous effect of the elastomeric infusion pump as well as the intraoperative periarticular injection of ropivacaine contribute to the effect of the cLIA method. This procedure was successfully applied as analgetic regimen after TKA for primary osteoarthritis [25] and adapted for our study design. The application of exclusively intraoperative periarticular injection of local anaesthetics was successfully implemented in TKA in general and in fast‐track concepts in particular [1, 27], but it omits the continuous effect by administering local anaesthetics per elastomeric infusion pump. Several study groups [25, 32, 42, 46] could demonstrate the efficacy of equivalent cLIA analgetic regimens for the treatment of postoperative pain after TKA for primary osteoarthritis. The aim of the recent study was to evaluate its value in the context of complex posttraumatic cases. The efficacy of exclusive periarticular infiltration analgesia without continuous application per elastomeric infusion pump in the context of TKA for posttraumatic arthritis finally remains doubtful but is subject to further research dealing with this issue.

PCIA outperformed cLIA regarding rescue analgesic use, though not significantly in terms of ROM or pain scores. However, interpretation is limited by potential bias: clinicians may hesitate to administer additional opioids to patients already on PCIA or may intervene more actively with less familiar methods such as cLIA. Future trials should account for provider behaviour to clarify true efficacy.

Notably, three deep infections occurred in the cFNB/cSNB group. Catheter‐side infections have to be differentiated from deep wound infections at the site of surgery, as they occurred in three cases in the present study. In 2023, Syrikas et al. published a systematic review on complication rates following TKA due to posttraumatic arthritis versus primary osteoarthritis [47]. The incidence of deep wound infections at the surgery site was increased in TKA for posttraumatic arthritis (range 1.9‐7.9%) compared with the primary osteoarthritis (range 0‐3%), with the difference reaching statistical significance in six studies. In the present study, three deep wound infections in the cFNB/cSNB group and no infections in both the pCIA and cLIA groups resulted in an overall infection rate of 3.3%. This rate is comparable to the results of the available literature in the setting of TKA for posttraumatic arthritis [7, 28, 47] but may raise concerns regarding the invasiveness and risk profile of perineural catheters. Published data from registries show a low overall incidence of peripheral catheter infections of <1% within the first four days of catheter retention, dramatically increasing with longer catheter retention times [5]. In the present study, cFNB/cSNB catheters were removed three days after the operation, so that the relatively high infection rate in the cFNB/cSNB cannot be explained by extensive retention times. Even more, these results emphasise the need for meticulous catheter care, especially in patients with prior trauma and compromised soft tissue [5, 9, 47].

Despite measurable differences in early function and pain, we found no significant group differences in the Knee Society Score (KSS) or overall patient satisfaction at discharge (t6). This may reflect the short observation period, as satisfaction and global functional scores often evolve over weeks to months. Prior studies suggest that meaningful differences in patient‐reported outcomes emerge only at mid‐ or long‐term follow‐up [24]. A re‐evaluation at six or twelve months postoperatively is warranted.

In the present study, the mean inpatient treatment period was 13.2 ± 5.3 days, which appears to be relatively long compared to the standard orthopaedic clinic and to institutions applying fast‐track rehabilitation protocols for TKA. Concordantly, our findings challenge the applicability of fast‐track protocols in the posttraumatic setting in TKA. Despite the theoretical advantages of cLIA in preserving muscle strength and earlier mobilisation [25], the higher pain burden and surgical complexity in posttraumatic cases appear to outweigh these benefits. As a result, early mobilisation remains limited regardless of the analgesic strategy, with possible advantages for the application of regional analgetic regimens like cFNB/cSNB, as shown in the present study. These observations are consistent with reports of prolonged recovery and higher resource utilisation in posttraumatic TKA [12, 49]. Recommendations for an optimised recovery of patients who underwent TKA for posttraumatic arthritis ‐ as they can be deducted from the results of the present investigation ‐ include the application on regional analgetic regimens like cFNB/cSNB, identification of patients at risk for higher postoperative pain levels due to their past medical history and preparation of both patients and surgeons for longer and possibly more complicated inpatient treatments and postsurgical rehabilitation periods.

### Limitations

The heterogeneity of trauma mechanisms, prior interventions, and surgical techniques poses a limitation to external validity. Another limitation is the choice of different levels of constraint in terms of an individualised surgical approach, without subgroup analysis due to small subgroup numbers. Additionally, rescue analgesic use was not blinded and may have been subject to provider bias. Consequently, the higher demand for rescue analgesics in cLIA than in PCIA must be interpreted with caution. The short‐term follow‐up limits conclusions about long‐term differences in joint function or patient quality of life, although early functional results are known to have an impact on the long‐term outcome of TKA. Nonetheless, the study's prospective design, robust randomisation, and focus on a highly specific and clinically relevant population are key strengths.

## CONCLUSIONS

In patients undergoing TKA for posttraumatic arthritis, cFNB/cSNB provided the most effective early postoperative pain relief and superior functional outcomes compared to cLIA and PCIA. As this result is limited to the early postoperative phase due to the study design and thereby must be interpreted carefully, a certain predictive value for the long‐term outcome of TKA for posttraumatic arthritis can be assumed. Furthermore, a higher complication risk in terms of deep wound infections must be taken into consideration. Given the complexity of this patient group, tailored analgesic strategies, balancing efficacy and safety, are essential. Further research should evaluate the long‐term impact of early analgesia on patient outcomes and investigate strategies to optimise recovery in this distinct population.

## AUTHOR CONTRIBUTIONS

BM and TO planned the study, were involved in data acquisition and analysis, and wrote the manuscript. BM, NB, AT and CS collected and analysed the data. CS, TO and CG carried out statistical analyses and produced the graphs. CGE, NB and CS were major contributors in writing the manuscript. TO, NB and MD supervised the study. All authors read and approved the final manuscript.

## CONFLICT OF INTEREST STATEMENT

The authors declare that they have no competing interests.

## ETHICS STATEMENT

The protocol was reviewed and approved by the ethics committee of the University of Witten/Herdecke (No. 19/2016) and did not change during the study. The informed consent was given by each patient included in this study. This investigation was conducted in accordance with the consolidated standards of reporting trials (CONSORT) guidelines and the principles of Good Clinical Practice. The trial was registered at the German Clinical Trials Register (No. DRKS00012500).

## Data Availability

The datasets used and/or analysed during the current study are available from the corresponding author on reasonable request.
